# The first mitochondrial genome data of an old world fruit bat, *Cynopterus sphinx* from Malaysia

**DOI:** 10.1080/23802359.2020.1846472

**Published:** 2021-01-12

**Authors:** Puteri Nur Syahzanani Jahari, Shahfiz Mohd Azman, Kaviarasu Munian, Nur Alwani Zakaria, Mohd Shahir Shamsir Omar, Stine R. Richter, Faezah Mohd Salleh

**Affiliations:** a Department of Biosciences, Faculty of Science, Universiti Teknologi Malaysia, Johor, Malaysia; b Forest Biodiversity Division, Forest Research Institute Malaysia, Kepong, Malaysia; c Faculty of Applied Sciences and Technology, Universiti Tun Hussein Onn Malaysia, Pagoh Higher Education Hub, Muar, Malaysia; d Section for Evolutionary Genomics, The GLOBE Institute, University of Copenhagen, Copenhagen, Denmark

**Keywords:** Cynopterus sphinx, species-complex, genetic variability, phylogenetic analysis

## Abstract

We assembled the complete mitogenome of *Cynopterus sphinx* (Vahl, 1797) of the family Pteropodidae originating from Malaysia. The total mitogenome size was 16,710bp which consists of 37 genes (13 protein-coding genes, 22 transfer RNA genes, two ribosomal RNA genes and one control region). A phylogenetic and BLASTn result showed the mitogenome sequence in this study varies by nearly 7% (93.48% similarity) from the same species in Cambodia. The next closest match of BLASTn was at 92% similarity to the *C. brachyotis*. This suggests the species-complex in *Cynopterus* sp. has given rise to the genetic variability.

Fruit bats play significant roles in the evolution and conservation of tropical forest ecosystems as they help in dispersing seeds and pollination of more than 300 plant species (Vanitharani et al. 2011). *Cynopterus* fruit bats are widely distributed in Southeast Asia, also known for its species-complex which comprises *C. sphinx*, *C. brachyotis* and *C. horsfieldi*. This is reported due to the combination of multiple factors such as past and present environmental changes and interspecific interactions (Campbell et al. 2006). Generation of complete mitogenomes for these species could be useful resources for future study to understand the relationship within the *Cynopterus* species-complex. In this study, we sequenced and provided the first whole mitochondrial genome of *C. sphinx* originating from Malaysia.

The specimen (voucher no: MZF01967) was collected from Temenggor, Grik, Perak, Malaysia (5.52 N 101.35 E) in January 2018 (Shahfiz et al. 2019) and currently stored in Zoological Collection of Forest Research Institute Malaysia (FRIM). Genomic DNA was extracted using Qiagen Blood and Tissue Kit (Qiagen, Valencia, CA). The DNA was later fragmented (300–400bp) using a M220 Focused-ultrasonicator (Covaris, USA) and BGISeq compatible shotgun sequencing library was built using the Blunt-End-Single-Tube (BEST) library protocol (Carøe et al. 2018; Jahari, Abdul Malik, et al. 2020; Jahari, Mohd Azman, et al. 2020). The library was pooled to equimolar and shotgun sequenced on BGISEQ-500 platform in 100 bp paired-end mode (PE100) (BGI, Shenzhen, China). Raw reads were trimmed for sequencing adapters, low-quality stretches, and leading/tailing Ns using AdapterRemoval v2.2.2 (Schubert et al. 2016). Forward and reverse reads were then interleaved into a single file. The assembly was conducted using MITOBIM v1.8 (Hahn et al. 2013) (default k-mer size of 31), which performs reference assemblies using MIRA iterations (Chevreux 2007). The mitogenome was annotated using the MITOS web server (Bernt et al. 2013).

The mitogenome of *C. sphinx* (MT259587) generated is a circular molecule with 16,710 bp in length. Similar to other *Cynopterus* species, it contained 13 protein-coding genes (PCGs), 22 transfer RNA genes, 2 ribosomal RNA genes and 1 control region (Yoon et al. 2016). The overall nucleotide of C. *sphinx* in this study reported having 32.02% A, 25.60% T,14.39% G and 28.00% C, which showed a slight AT bias (57.62%), similar to other vertebrate mitogenomes (Yoon et al. 2016; Han et al. 2019). The total length of the protein-coding gene sequences (PCGs) is 11,404 bp. The total length of the 22 tRNA genes is 1506 bp, ranging from 57 bp (tRNASer) to 79 bp (tRNAGln). The 12S rRNA gene length is 964 bp and the 16S rRNA gene length is 1565 bp, and are located between the tRNAPhe and tRNALeu, and are separated by the tRNAVal gene. The control region is located between tRNAPro and tRNAPhe genes. The genes mostly located on the heavy (H) strand except for NAD6 and eight tRNAs genes (tRNAGln, tRNAAla, tRNAAsn, tRNACys, tRNATyr, tRNASer, tRNAGlu, tRNAPro), which were found to be located on the lower (L) strand.

The BLASTn result showed the highest hit matched by 93.48% to the same species, *C. sphinx* (NC_046902.1) from Cambodia (Hassanin et al. 2020), followed by *C. brachyotis* (MN816304.1) (Hassanin et al. 2020) at 92% and another sequence of the same species (KM659865.1) originating from a different state of Malaysia, Selangor Darul Ehsan (Yoon et al. 2016). Phylogenetic trees of all available *Pteropodinae* subfamily mitogenomes was constructed using the neighbor-joining (NJ), maximum-likelihood (ML) and Bayesian method. A mitogenome of a hedgehog, *Erinaceus europaeus* (accession no. NC_002080.2) was selected as an outgroup. The phylogenetic tree generated a monophyletic clade of *Cynopterus* species which includes *C. sphinx* and *C. brachyotis* at highly supported bootstrap values. According to Campbell et al. 2004, *C. sphinx*, *C. brachyotis* and *C. horsfieldi* are mostly distributed members of the *Cynopterus* genus that have complex distinct evolutionary lineages. The recent evolutionary histories between these *Cynopterus* sp. was shaped by interaction between multiple factors including environmental changes (Campbell et al. 2006). Besides, the overlap morphological features between species representatives across geographical gradients has also challenged the species identification of *Cynopterus* sp. (Jayaraj et al. 2012). It is clear therefore, future studies on comparative mitogenomes will unfold the mystery of the origin and spread of this *Cynopterus* sp. across its range (Figure 1).

**Figure 1.**
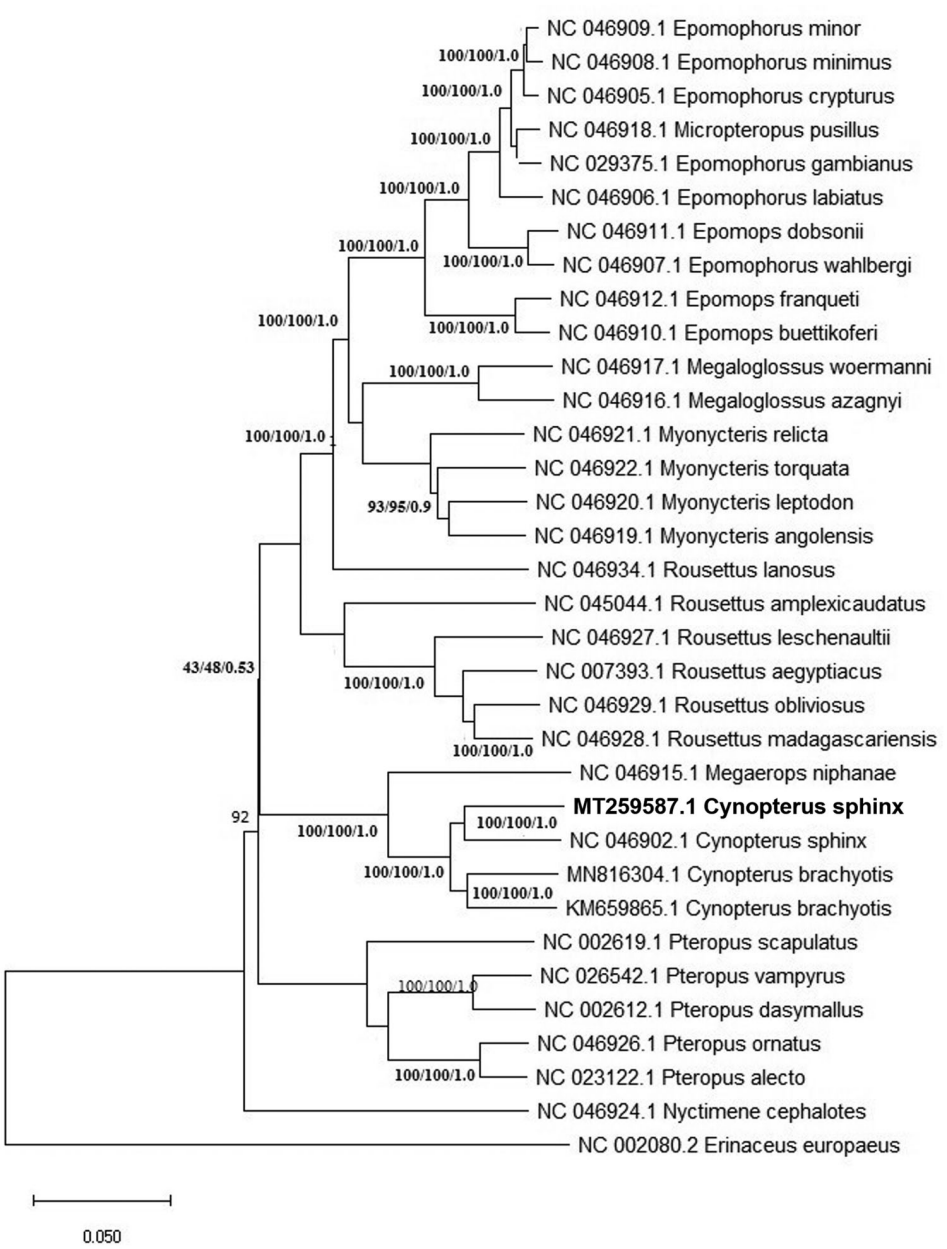
The phylogenetic tree of *C. sphinx* was constructed based on present mitogenome (MT259587) and other curated *Pteropodinae* subfamily-derived species available in Genbank. A mitogenome of a hedgehog, *Erinaceus europaeus* (accession no. NC_002080.2) was selected as an outgroup. Bootstrap values were indicated in each branch of the tree representing the result of NJ/ML/Bayesian probability.

## Data Availability

Mitogenome data supporting this study are openly available in GenBank at: https://www.ncbi.nlm.nih.gov/nuccore/MT259587. Associated BioProject, SRA, and BioSample accession numbers are https://www.ncbi.nlm.nih.gov/bioproject/PRJNA610427, https://www.ncbi.nlm.nih.gov/sra/SRR11241208, and https://www.ncbi.nlm.nih.gov/biosample/SAMN14297803, respectively.
